# Study protocol: an early intervention program to improve motor outcome in preterm infants: a randomized controlled trial and a qualitative study of physiotherapy performance and parental experiences

**DOI:** 10.1186/1471-2431-12-15

**Published:** 2012-02-15

**Authors:** Gunn Kristin Øberg, Suzann K Campbell, Gay L Girolami, Tordis Ustad, Lone Jørgensen, Per Ivar Kaaresen

**Affiliations:** 1 Faculty of Health Sciences, Department of Health and Care Sciences, University of Tromsø, 9037 Tromsø, Norway; 2 Faculty of Health Sciences, Department of Clinical Medicine, University of Tromsø, 9037 Tromsø, Norway; 3 Clinic of Rehabilitation, Physical Therapy Section, University Hospital of Northern Norway HF, 9038 Tromsø, Norway; 4 BUK, University Hospital of Northern Norway HF, 9038 Tromsø, Norway; 5 Clinic of Clinical Services, University Hospital Trondheim, St.Olavs Hospital HF, 7006 Trondheim, Norway; 6 University of Illinois at Chicago, Chicago, USA

**Keywords:** Preterm infants, early intervention, Physiotherapy, Motor development, Parental experience

## Abstract

**Abstract:**

**Background**

Knowledge about early physiotherapy to preterm infants is sparse, given the risk of delayed motor development and cerebral palsy.

**Methods/Design**

A pragmatic randomized controlled study has been designed to assess the effect of a preventative physiotherapy program carried out in the neonatal intensive care unit. Moreover, a qualitative study is carried out to assess the physiotherapy performance and parents' experiences with the intervention. The aim of the physiotherapy program is to improve motor development i.e. postural control and selective movements in these infants. 150 infants will be included and randomized to either intervention or standard follow-up. The infants in the intervention group will be given specific stimulation to facilitate movements based on the individual infant's development, behavior and needs. The physiotherapist teaches the parents how to do the intervention and the parents receive a booklet with photos and descriptions of the intervention. Intervention is carried out twice a day for three weeks (week 34, 35, 36 postmenstrual age). Standardized tests are carried out at baseline, term age and at three, six, 12 and 24 months corrected age. In addition eight triads (infant, parent and physiotherapist) are observed and videotaped in four clinical encounters each to assess the process of physiotherapy performance. The parents are also interviewed on their experiences with the intervention and how it influences on the parent-child relationship. Eight parents from the follow up group are interviewed about their experience. The interviews are performed according to the same schedule as the standardized measurements. Primary outcome is at two years corrected age.

**Discussion**

The paper presents the protocol for a randomized controlled trial designed to study the effect of physiotherapy to preterm infants at neonatal intensive care units. It also studies physiotherapy performance and the parent's experiences with the intervention.

**Trial registration:**

ClinicalTrials.gov NCT01089296

## Background

Preterm children are at increased risk of motor impairments and these impairments often persist into adolescence [1]. Evidence regarding the effect of physiotherapy to improve motor development in preterm infants is limited [2]. Interventions designed for promoting development in these infants have been heterogeneous and studies reporting a significant impact of early intervention on motor development are sparse [2,3]. Examining an approach in which the therapy is adapted to the individual premature infant's needs may contribute to knowledge about how to enhance motor development in these infants. To that end we designed a study on the effects of physiotherapy in infants born prematurely as well as on professional performance and parents experiences. The intervention is performed before the infant's reach term age.

The study, named "The Norwegian Physiotherapy Study in Preterm Infants" (NOPPI), consists of a pragmatic randomized controlled trial and a qualitative observational and interview study. The project provides a new approach to intensive physiotherapy consisting of several more elements than today's traditional approach. The intervention integrates key elements from the modified version of the Mother-Infant Transaction Program performed in a study by Kaaresen and colleagues [4,5], as well as elements from interventions in other studies which have shown a positive effect on premature children's motor development [2,3,6-9]. NOPPI explores the effects of individually customized physiotherapy on preterm infants before they reach term age as well as assess the physiotherapy performance and parental experiences of participating in carrying out the intervention in the neonatal intensive care unit (NICU). Outcomes are measured up to two years of age.

The theoretical framework related to the physiotherapy intervention in this study is knowledge of newborn behaviors [10,11], the importance of parental competency [5,12] and theories of motor development, including neuroscience and phenomenology of the body [13-15]. A brief presentation of the framework follows.

### Newborn behaviour and parental competency

Competency in behavioral organization makes active social participation possible for infants [10,11]. As a group, however, prematurely born infants with very low birth weight, and particularly those with serious complications, are reported to have more difficulties in behavioral regulation than infants born at term [16,17]. This may be expressed by the infant as irritability, requiring a long time to settle into a routine and fluctuating attention. Infants' neurobehavioral functioning unfolds through maturation and experience, and the individual can be helped to self-regulate by the caregiver and environmental adaptations. Parental competency to read and understand the individuality and needs of their infant is significant in decreasing parental stress [5] and enhances cognitive outcome and social functioning in the infants [18].

### Phenomenology of the body

The body forms the base from which both the infant as a person and the world are constituted. A newborn's body is a tactile-kinesthetic body. Through moving, infants learn and experience movements by which kinesthetic competency develops [19,20]. On the basis of innate spontaneous movements, the infant learns to know their own body as well as gaining knowledge and realization of the surroundings. Their bodies are both expressive and experienced at the same time. Thus, child development can be understood as a result of interaction among the system consisting of perception, sensation and movement.

### Theory of motor development

The motor development of a child is non-linear [21,22] and regarded as a product of both genetic processes and experiences [23,24]. In dynamic systems theory [25], motor development is believed to be a feedback process based on interaction among different subsystems in the child, the environment and the task. There is a shift from trial and error phases of instability to stable movement in which the synergy of appropriate movements is used to perform a functional task [23]. The motor patterns of healthy children appear flexible, adaptable and dynamic [23].

The motor patterns of preterm infants are dominated by extension and to a lesser degree flexion when compared to infants born at term [26]. This fact, in addition to possible brain damage, may influence the children's spontaneous motor experiences and the process of developing stable motor strategies as they grow. Motor function is related to the development of postural control which is necessary to transfer and modify body weight distribution for appropriate functional movement, communication and social interaction [27,28]. To have postural control is then about maintaining a bodily position over time, regaining postural stability after perturbations, managing changes between different postures, and integration of postures into locomotion and exploration [27]. Interventions that optimize postural control and selective movement in preterm infants may therefore be important in reducing the degree of delayed motor development or the severity of cerebral palsy (CP).

The human brain in infancy is highly plastic and there is an active growth of dendrites and formation of synapses. Experience influences and models the brain and leads to structural changes [24,29] in, e.g., the number of synapses that are developed, the synapses' position and functioning, as well as elimination of synapses that are not needed. Motor skills may be highly influenced by early intervention because the motor pathways forming the corticospinal tracts already show mature myelin at term age [30] and myelination may be activity-dependent [31].

There is some evidence that recovery from central nervous system injury in infants can be understood both by new growth of motor neurons and creation of new synapses. Moreover that part of the brain is not yet developed for specific tasks and may be developed for other uses than were originally intended [24]. Of these insights about brain plasticity it is suggested that early-targeted customized individual intervention could be of great importance to the development of movement quality and function of preterm children.

## Methods/Design

NOPPI consists of two related parts. The aim of the first part, the pragmatic randomized controlled trial, is to evaluate the effect of customized physiotherapy on preterm infants' motor development when the intervention is performed by the parents during a period of three weeks while the infant resides in the NICU. The endpoint is motor development at 24 months of corrected age (CA).

The aim of the second part, the qualitative observation and interview study, is one: to analyze and identify aspects of physiotherapy performance important for teaching parents practical knowledge, and two: to increase our knowledge about parents' experiences of active involvement in implementation of the intervention designed to promote their child's motor development, as well as the short and long term effects on the parent-child relationship. The endpoint is 24 months CA.

The study is approved by the Ethic Committee of Northern Norway (REK nord: 2009/916-7).

### Part one

#### Study sample

Prematurely born infants at the University Hospital Northern Norway HF, Tromsø, Norway, and University Hospital Trondheim HF, St. Olavs Hospital, Norway, with gestational age (GA) at birth ≤ 32 weeks are eligible for the study. The infants must be able to tolerate handling at postmenstrual age (PMA) week 34 and their parents have to understand/speak Norwegian. In addition it is required that the follow-up program takes place at the respective hospitals outpatient clinics. Exclusion criteria are triplets or higher plurality, major malformations or recent surgery.

#### Sample size calculations

Power calculation was performed. Our outcome measure at 24 months CA is the Peabody Developmental Motor Scales-2 (PDMS-2) [32]. We consider a difference on gross motor and fine motor function measured on PDMS-2 between the intervention and the control group of 0.5 SD as clinically significant. As a result there must be 63 children in each group to have an 80% chance to detect a 0.5 SD difference between the groups with a significance level of 0.05 (alpha) on two-sided tests. When we consider potential attrition and the effect of including twins, we aim to recruit 150 children, i.e., 75 in each group for part one of the study.

#### Recruitment procedure

Enrollment of participants is a process taking place at the neonatal units of two Norwegian University Hospitals. Oral and written information is given to parents of the preterm babies fulfilling the inclusion criteria. Professionals not involved in the daily care and treatment of the child when the child is 33 weeks PMA conduct the interview. It is the project leader who performs the recruitment interview in Tromsø, while the representative in the project leader group in the other Hospital (St. Olavs Hospital, Trondheim) addresses the parents in Trondheim. Informed consent forms signed by the parents are delivered to a nurse or physiotherapist in the neonatal unit if the parents agree to participate, after which the baseline assessment is performed.

#### Randomization process

**Figure 1:**
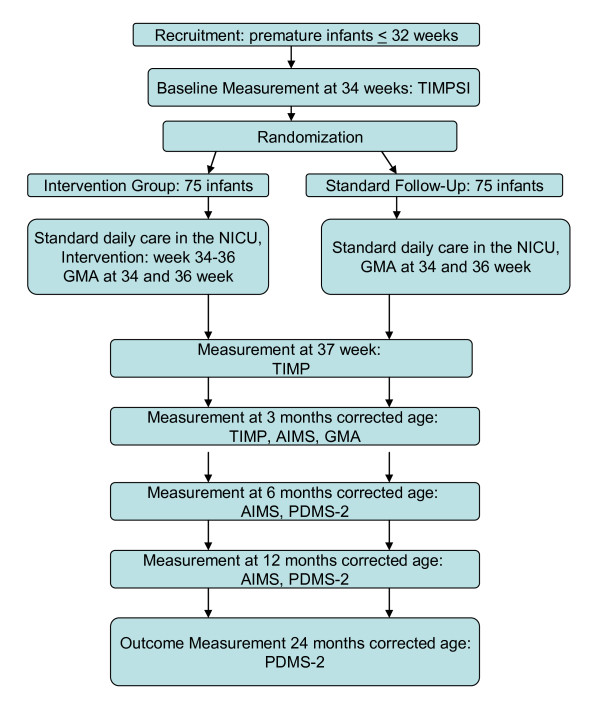
**Flowchart of the quantitative study, part one**.

The infants are randomly assigned either to the intervention or to the control group. Randomization is performed by a web-based randomization system developed and administered by the Unit of Applied Clinical Research, Institute of Cancer Research and Molecular Medicine, Norwegian University of Science and Technology, Trondheim, Norway. Stratification is according to GA at birth (< 28 week and ≥ 28 weeks) and recruitment site. In the case of twins both children are randomized to the same group because of the nature of the intervention. The randomization takes place *after *the assessment of baseline motor performance (Figure 1) so that the therapists will not be biased one way or the other by knowing the group assignment.

#### Intervention

##### Practitioners

Experienced physiotherapists in pediatrics are implementing the intervention and perform the assessments. In each research centre two physiotherapists are dedicated to performing the baseline assessment and teaching the treatment protocol to the parents of the intervention group infants. Each therapist maintains records (log) over the number of clinical consultations with the individual child and parent and notes what has been emphasized in the consultations. Two other physiotherapists blinded to group assignments perform the follow up assessments when the child is at term and at three, six, 12 and 24 months CA. The physiotherapists are assessed for rater reliability for the standardized tests used.

##### Content of intervention

The intervention involves education of parents in individualized handling and motor stimulation of their child. The handling and motor stimulation program is primarily based on Girolami and Campbell [6], and the performance is integrated into communication and social interaction between the caregiver and the infant [5]. The parent at the bedside of the child during the NICU admission period is the one carrying out the daily intervention after being taught by the physiotherapist. The objective of the intervention in which the main elements are postural support and movement facilitation techniques, is on improving symmetry of posture, muscle balance, and movement in infants, all of which are supporting the foundation of the execution of functional activities in the infant's daily life. The facilitating technique is intermittent adjusted pressure/compression over relevant muscle groups and joints when the infant is in supine (Table 1), prone (Table 2), sidelying (Table 1) and in supported sitting (Table 2). There are also transition activities in which the infant is guided from supine to sidelying and from supine through sidelying to upright supported sitting (Table 1). The physiotherapist chooses appropriate exercises and modifies handling for each infant's level of development and tolerance for movement; the intervention always includes one or more activities in each position. A main goal is development of head and trunk control in each position.

**Table 1:** The protocol for promotion of postural and selective control of movements, supine and sidelying

Objectives	Performer activity	Activity goals for the child
1. Increase strength, balance. Control of the anterior and posterior neck muscles.	1. Activating neck flexors, shoulder and abdominal muscles through intermittent caudal compression.	1. Maintain head in midline and head turning to both sides.
2. Increase strength and control of the anterior shoulder and chest muscles and balance between anterior and posterior shoulder and chest muscles.	2. Horizontal intermittent pressure through the shoulders. Assist the child to bring arms forward to the mouth or on chest.	2. Bringing hands forward, hands to mouth and hands on chest.
3. Increase strength and control of the abdominal muscles.	3. Through lifted pelvis and flexed legs, provide intermittent compression toward shoulder.	3. Antigravity pelvis and lower extremity lifting with hip and knee flexion
4. Affect alignment, righting reactions and antigravity muscle activity in the trunk in the sagital and frontal planes.	4. From the lifted pelvis and control at shoulders, shift the infant's weight in small increments from side to side. When possible allow the infant to control the head and arms without assistance.	4. Rolling from supine to side.
5. Affect alignment, righting reactions and balance and control between the anterior and posterior neck and trunk muscles.	5. Guide the child from supine through sidelying to upright sitting.	5. Maintaining head control in midline during the transition with minimal assist.
6. Increase strength of the anterior neck muscles lateral head righting and neck and cervical extensors when rolling into prone.	6. Guiding upper shoulder slightly backwards with small weight shifting movements while supporting the child with one hand under head.	6. Keep the chin tucked during movements from supine to prone and when in sidelying
7. Increase the strength of the anterior chest and shoulder muscles.	7. Horizontal intermittent compression through the shoulders. Assist the infant in bringing the hands to mouth or toward the midline.	7. Bring hands to mouth or bring hands forward to chest.
8. Elongation of thorax and lumbar muscles; increase strength, balance and control of abdominal and trunk muscle groups.	8. Lifting pelvis laterally upward to lengthen the weight-bearing side of trunk and activate lateral muscles of the trunk and head on the non-weight-bearing side. Facilitate rolling from supine to side. Head, neck, trunk and pelvis are in alignment.	8. Maintain the pelvis in a neutral position while flexing the hip and knee. Improved antigravity strength of the lateral neck and trunk muscles

1-5: The child is in supine. 6-8: The child is sidelying

**Table 2:** The protocol for promotion of postural and selective control of movements, prone and sitting

Objectives	Performer activity	Activity goals for the child
1. Increase strength, balance and control in the anterior and posterior neck and upper back muscles.	1. Intermittent compression through shoulders in caudal direction is used to activate the neck muscles, pectoralis muscles and upper back extensors.	1. Lifting the head from the surface and turning the head to right and left side.
2. Increase strength and balance of the anterior and posterior shoulder muscles.	2. Mild intermittent horizontal compression through shoulders to activate the anterior and posterior shoulder and scapular muscles.	2. Bring the hands to mouth.
3. Downward rotation and stabilization of the scapula.	3. Small weight shifts to one side to facilitate head turning by providing compression down the non-weight-bearing side and elongation of the weight-bearing side.	3. Strength and control of shoulder girdle to provide a stable base for head lifting and turning.
4. Increase activity and strength of the abdominal muscles.	4. Support and tactile input over the abdominal muscles to increase activation in the sagital and frontal planes.	4. Maintain the pelvis in neutral to provide stable base of support for trunk extension and sagital and frontal plane weight shifts.
5. Increase strength and control of neck muscles; elongation of cervical spine.	5. Intermittent compression through the shoulders in a caudal direction to facilitate balanced activation of the anterior and posterior neck, chest and abdominal muscles.	5. Maintain the head up and in midline.
6. Increase strength, balance and control of anterior and posterior neck muscles and downward rotation of the scapula.	6. Intermittent horizontal compression through shoulders and chest muscles to assist the infant to bring the hands together in midline or to the mouth.	6. Maintenance of scapular depression to assist in bringing hands to midline.
7. Integrate control of abdominal muscles and back extension muscles; increase the strength of abdominal muscles; improve balance of trunk flexor/extensor muscle activity.	7. Support the head and shoulders and tip the infant approximately 15 degrees backward to activate neck and abdominal muscles. From this position add very small lateral movements to activate trunk in the frontal plan, elongating the weight-bearing side of the body to promote lateral righting of the head and trunk.	7. Maintain capital flexion, chin toward the chest with hips and knees in neutral flexed position.

1-4: The child is in prone. 5-7: The child is in sitting

Functional goals and activities for the child in supine include: maintaining head in midline, rotating the head to right and left, bringing hands to mouth and hands to chest, adjusting their own position, turning from supine to side (Table 1). Sidelying activities include maintaining a comfortable position with head flexed toward chest, bringing hands to mouth (Table 1). Prone activities include assisting the infant to lift and turn the head to the middle and to right and left sides, adjust their position, take weight on forearms, bring the hands to the mouth, look for the caregiver (Table 2). Finally, supported sitting activities include maintaining controlled upright and midline posture of the head with good trunk extension, being able to turn the head to track and using the arms for forward reaching (Table 2).

Intervention is carried out for up to ten minutes, twice a day, over a period of three weeks (PMA weeks 34, 35, 36). During intervention the infant should be in "State of arousal level" three (eyes open, no movements) or four (eyes open, large movements) according to Prechtl's states [33]. The length of each treatment session is adjusted depending on the infant's response and condition. Intervention is terminated if the infant shows any of the following signs which are interpreted as expressions of stress or discomfort: makes faces, changes skin color, has irregular respiration, undesired changes in muscle tone, uncontrolled movements or continual changes in the state of arousal level. Performance time is adjusted to the infant's daily rhythm. Intervention may be carried out half an hour before a meal, between two meals or any time when the child has a state of arousal level of three or four. Parents record the time of each intervention and the number of interventions each day. If necessary they note concisely why intervention was not completed. At the very beginning of the intervention period parents receive a "play book" in which they find pictures and written explanations of each "exercise" they will be performing during the intervention period. The parents have to demonstrate their ability to do the activities the second and the eighth day of the intervention.

##### Test instruments

Demographic data as well as information about current diseases are collected from patient records, from the NICU's online registration program and by interviewing the parents. All infants participating in the study are assessed with standardized tests at term age, three, six, 12 and 24 months CA (Figure 1). Motor development at baseline is assessed using the Test of Infant Motor Performance Screening Items (TIMPSI) at 34 weeks PMA. The TIMPSI addresses the main targets for the intervention, postural control and selective movements. The primary outcome measure is motor development at two years CA on the Peabody Developmental Motor Scales (PDMS-2). The PDMS-2 was chosen because the test assesses both fine and gross motor function, i.e., harmonizing with the intervention targets of postural control and selective movements. The PDMS-2 is also administered at six months and 12 months CA (Figure 1). Secondary outcome measures are: the General Movement Assessment (GMA) at 34 weeks, 36 weeks, and three months CA, the Test of Infant Motor Performance (TIMP) at 37 weeks, and three months CA, and the Alberta Infant Motor Scale (AIMS) at three months, six months, and 12 months CA (Figure 1).

##### Test of Infant Motor Performance Screening Items

Scores on the Test of Infant Motor Performance Screening Items (TIMPSI) form the baseline for assessment of each infant's motor performance prior to initiation of the intervention. The TIMPSI assesses movement and postural control in prone, supine, and supported sitting and standing and takes approximately 20 minutes to administer [34]. The TIMPSI is composed of three subsets of items taken from the Test of Infant Motor Performance (see next paragraph). Prior to assignment to one of the TIMPSI subsets, TIMP items were psychometrically analyzed using Rash analysis. The first set of eleven items, representative of the full TIMP, is administered. Based on the infant's score, either an "easy set" (ten items) or a "hard set" (eight items) is administered [34]. The test results are used in the ultimate statistical analysis of results as well as to determine the emphasis of the treatment protocol.

##### The Test of Infant Motor Performance

The Test of Infant Motor Performance (TIMP) identifies age-appropriate or delayed motor development in infants and shows changes in motor development with increasing age [34]. The test evaluates postural control-stability and alignment of parts of the body - in addition to the child's reactions to visual and auditory stimuli. The TIMP is valid for use from 34 weeks PMA until five months CA. The test consists of 13 Observed Items and 29 Elicited Items [34]. Previous studies have demonstrated that the TIMP is responsive to intervention in preterm infants both prior to term age [6] and from term to four months CA [35]. The age of testing is best at approximately the same time within normative windows for all children in the study, i.e., the test is performed as close to the middle of the two-week age window as possible.

##### Prechtl's Method of General Movement Assessment

Prechtl's Method of General Movement Assessment (GMA) identifies normal and abnormal quality of movement (CP)[36]. The GMA is valid for use from preterm age until about five months CA. The scoring, based on taped observation of spontaneous movement recorded while the infant is supine, is considered to be a non-invasive assessment because no handling is involved. Recommendations for the recording technique [36] include video recordings from five to thirty minutes in duration depending on the age and activity level of the infant. General Movements are first clearly defined as either normal movement patterns or abnormal ones, following which abnormal General Movements are classified in different subgroups dependent of the infants age [36]. The subgroup at the age of 34 and 36 PMA are Poor Repertoire (PR), Cramped-Synchronized (CS) and Chaotic (CH) General Movements. At three months there is No Fidgety (F-) or Abnormal Fidgety Movements (AF). Both the TIMP and the GMA are used for concurrent assessment at term and three months CA because at term age they have been shown to predict different aspects of development at one year of age, i.e., TIMP scores are related to functional performance and the GMA to locomotion at one year [37]. The GMA has high sensitivity and specificity for the prediction of CP by three-four months CA [38,39].

##### Alberta Infant Motor Scale

The Alberta Infant Motor Scale (AIMS) examines delayed and abnormal motor development in infants over time and is valid for assessment from term until 18 months of age [40]. The test, selected because of good psychometric properties, is quick to administer with limited handling and focuses on both achievement of motor milestones and quality of posture and movement outcomes [41]. The age of testing is done at approximately the same time within the one-month normative window for all children at three, six and 12 months CA, i.e., the test is performed as close to the middle of the age window as possible. Pin and colleagues [42] demonstrated the sensitivity of the AIMS items to differences in preterm infant motor development that typically result in lower scores for preterm than for full term infants [32].

##### Peabody Developmental Motor Scales

The Peabody Developmental Motor Scales (PDMS-2) assesses both fine and gross motor function [32]. The test is valid from term through five years of age. PDMS-2 consists of six subtests e.g. Reflexes, Stationary, Locomotion, Object Manipulation, Grasping and Visual-Motor Integration. The results of the subtests may be used to generate three global indices of motor performance. These composites are Gross Motor Quotient, Fine Motor Quotient and Total Motor Quotient [32]. The three composites of the PDMS-2 exhibit high test-retest reliability and acceptable responsiveness to intervention effects [43]. The test is suitable to use as a motor measure for children with CP at two years of age [43].

##### Data collection

Both the intervention group and the control group receive standard medical and nursing care while hospitalized. The Newborn Individualized Development Care and Assessment Program (NIDCAP) [44,45] forms the principal approach in the NICU. In addition the intervention group receives the handling and facilitation program. The nurses are not blinded for the group assignment because it is impossible to prevent them from observing the parents providing the intervention protocol. However, we discussed prior to the initiation of the study the need to refrain from applying the intervention to any infants in the NICU.

After discharged from the hospitals, infants from both groups return for the follow up at the Hospitals' outpatient clinics. If the pediatrician and the physiotherapist assessing the infant judge additional physiotherapy to be needed after discharge, individuals will be referred to therapy independent of group assignment. The physiotherapist in the outpatient clinic records information if infants receive physiotherapy after discharge from the Hospital.

##### Analysis

Demographic data will be collected and described with descriptive statistics. Group differences will be analyzed using linear mixed models for continuous data and generalized estimating equations (GEE) for categorical data. These methods make it possible to account for the possible clustering effect by including twin pairs and for repeated measurements. Z-scores will be used in the longitudinal analyses as different tests are used, as the child gets older. All the tests are double sided tests and *p*-value < 0,05 is considered significant. SPSS and Stata will be used in the analyses.

##### Data storage

Test results are recorded on original test forms and stored safely. The results are entered into a secure research database at the University Hospital of Northern Norway using the statistical program SPSS.

### Part two

#### Study sample

Part two involves a qualitative study based on a subset of subjects from the clinical trial: eight triads (physiotherapist, parent and infant) from the intervention group and parents of eight infants in the control group.

#### Recruitment procedure

Parents of infants from the intervention and from the control group are invited to participate in the qualitative study. Recruitment is an ongoing process until we have the planned number of sixteen participants.

#### Design

**Figure 2:**
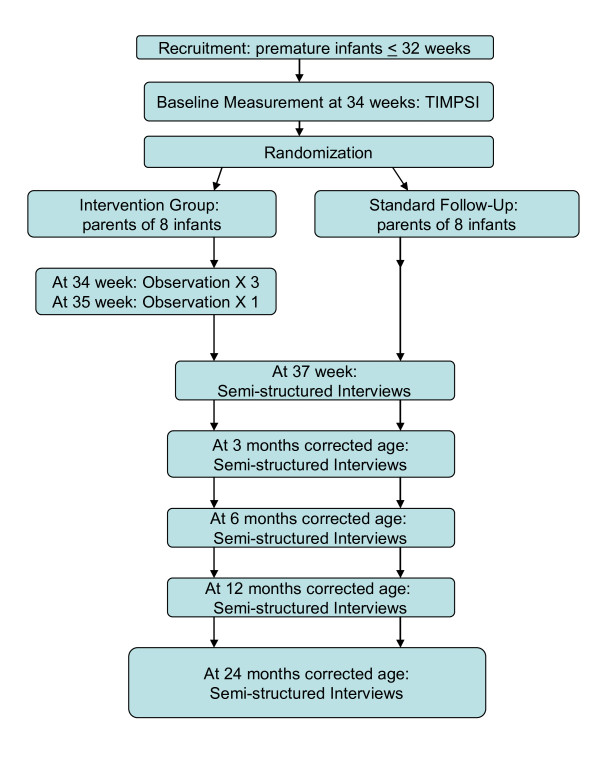
**Flowchart of the qualitative study, part two**.

Part two of the study has an exploratory design [46]. Because the objective is both to increase knowledge about physiotherapy performance and to increase the understanding of parents' experience of being actively involved in implementation of the intervention, as well as the effects on the parent-child relationship in short and long term, repeated observation and qualitative interviews are chosen as the research methods. The schedule for observations and interviews is described in Figure 2.

The observations of clinical encounters with participants from the intervention group focus on what is going on in the situation, i.e., communication and interaction between the parent and therapist, between the therapist and infant and between the parent and infant during therapy. The clinical encounters are videotaped. In addition there are qualitative semi-structured interviews with the caregivers from both groups. The themes in the interview guide include: feelings and observations about the infant, interplay and interaction with the infant. For the intervention group the topics also include parents' guidance and parents' reflections on cooperation with the physiotherapist and the experience of the intervention. There are open-ended questions.

##### The intervention group

• Observation and video recording of the TIMPSI in PMA week 34, parents present, the first two consultations after the TIMPSI and eight days after the last consultation in week 34.

• Interview with the parent who carries out the intervention: before discharge from hospital, and at follow up at three, six, 12 and 24 months CA. Interviews will be audio recorded.

##### The control group

• Interview with the parent who spends most time at the hospital with the child during the neonatal admission period for the eight children in this group. Interviews will be recorded and carried out before discharge from hospital, and at three, six, 12 and 24 months CA.

#### Observational and interview personnel

The project leader and the collaborating partner who is a member of the project leader team in Trondheim are doing the observations and the interviews in, respectively, Tromsø and Trondheim. Neither of the researchers are therapists for the infants and parents participating in the qualitative part of the study. Both researchers are physiotherapists, have been working in the field of pediatrics for several years, and are skilled in observation and interview techniques.

#### Data analysis

A phenomenological-hermeneutic analysis ad modum Lindseth and Norberg [47] will be carried out on the data material from the observations and interviews. The interpretation process will follow the hermeneutic circle from whole to part and part to whole. Steps in the process of analysis:

1. Each video clip is studied and the general impression is summarized.

2. Structural analysis of each situation. Identification of main theme and sub theme.

3. Description of main theme and sub themes.

4. Structural analysis is compared with the general impression from the video clips.

5. Revision and adjustment by repeating 1-4.

6. All the video clips with main theme and sub themes are studied in the same context.

7. A complete interpretation of the data is produced.

The same process of analysis is used for the transcripts of the interviews. Trustworthiness (credibility and dependability of the findings) will be established through triangulation of the deriving themes of two or three researchers.

## Discussion

This paper presents a health promoting individually customized physiotherapy program designed for preterm infants before they reach term age to improve the infants' motor development. The intervention program is based on current theoretical frameworks and includes aspects of previously successful interventions such as the significance of infants' behavioral regulation and parent competency in social interaction. The design is appropriate for implementation in a NICU setting, but may be feasible to pursue in a community setting and generalized across different groups of high risk infants. The Norwegian Physiotherapy Study in Preterm Infants provides an opportunity to determine whether an individually customized three-week physiotherapy program for preterm infants in the NICU, will enhance the infants' motor development at two years CA. The study will also provide insight into the process of communicating practical knowledge to parents and the value of parent's handling competency in interaction with the preterm infant. The study has both qualitative and quantitative elements.

## Competing interests

SKC and GLG are co-developers of the TIMP and partners in Infant Motor Performance Scales, IIC. The authors proclaim that there are no other conflicts of interests.

## Authors' contributions

GKØ conceived the study, designed it, and drafted the manuscript. PIK participated in the study design and coordination, and helped to draft the manuscript. TU participated in the conception and formulation of the study design. SKC were involved in the conception and design of the study. GLG were strongly involved in the design of the intervention package. TU, SKC, GLG and LJ provided critical review and all authors provided final approval of the draft. All authors read and approved the final manuscript.

## Pre-publication history

The pre-publication history for this paper can be accessed here:

http://www.biomedcentral.com/1471-2431/12/15/prepub
